# Phase I study of afatinib combined with nintedanib in patients with advanced solid tumours

**DOI:** 10.1038/bjc.2015.374

**Published:** 2015-10-29

**Authors:** Rastislav Bahleda, Antoine Hollebecque, Andrea Varga, Anas Gazzah, Christophe Massard, Eric Deutsch, Nadia Amellal, Françoise Farace, Mahmoud Ould-Kaci, Flavien Roux, Kristell Marzin, Jean-Charles Soria

**Affiliations:** 1 Drug Development Department, Gustave Roussy, 114 Rue Edouard Vaillant, 94805 Villejuif Cedex, France; 2 Drug Development Department and Radiation Therapy Department, Gustave Roussy, 114 Rue Edouard Vaillant, 94805 Villejuif Cedex, France; 3 Inserm Unit 981, Gustave Roussy, Villejuif Cedex, Paris, France; 4 Boehringer Ingelheim, 14 rue Jean Antoine de Baif, 75013 Paris, France; 5 Boehringer Ingelheim, Reims, France; 6 Boehringer Ingelheim Pharma GmbH & Co. KG, Biberach, Germany

**Keywords:** afatinib, nintedanib, ErbB family blocker, angiokinase inhibitor, angiogenesis, Phase I, circulating tumour cells

## Abstract

**Background:**

This Phase I study evaluated continuous- and intermittent-dosing (every other week) of afatinib plus nintedanib in patients with advanced solid tumours.

**Methods:**

In the dose-escalation phase (*n*=45), maximum tolerated doses (MTDs) were determined for continuous/intermittent afatinib 10, 20, 30 or 40 mg once daily plus continuous nintedanib 150 or 200 mg twice daily. Secondary objectives included safety and efficacy. Clinical activity of continuous afatinib plus nintedanib at the MTD was further evaluated in an expansion phase (*n*=25).

**Results:**

The most frequent dose-limiting toxicities were diarrhoea (11%) and transaminase elevations (7%). Maximum tolerated doses were afatinib 30 mg continuously plus nintedanib 150 mg, and afatinib 40 mg intermittently plus nintedanib 150 mg. Treatment-related adverse events (mostly Grade ⩽3) included diarrhoea (98%), asthenia (64%), nausea (62%) and vomiting (60%). In the dose-escalation phase, two patients had partial responses (PRs) and 27 (60%) had stable disease (SD). In the expansion phase, one complete response and three PRs were observed (all non-small cell lung cancer), with SD in 13 (52%) patients. No pharmacokinetic interactions were observed.

**Conclusions:**

MTDs of continuous or intermittent afatinib plus nintedanib demonstrated a manageable safety profile with proactive management of diarrhoea. Antitumour activity was observed in patients with solid tumours.

Cancer cells exhibit dysregulation of multiple signalling pathways that facilitate tumour growth and progression and often demonstrate a certain degree of cross-talk (Tabernero, 2007; Larsen *et al*, 2011). Members of the ErbB receptor family, including epidermal growth factor receptor (EGFR/ErbB1), human epidermal growth factor receptor (HER2/ErbB2), ErbB3 (HER3) and ErbB4 (HER4), have important roles in cell proliferation, migration and apoptosis, and have been implicated in a variety of malignancies (Yarden and Pines, 2012). Vascular endothelial growth factor (VEGF), a key regulator in angiogenesis, also has an important role in tumour growth and metastasis (Tabernero, 2007), and expression levels of VEGF and VEGF receptors (VEGFRs) have been correlated with poor outcomes in numerous tumour types (Goel and Mercurio, 2013).

Several agents that individually target the EGFR or VEGFR pathways have been approved for the treatment of cancer, particularly non-small cell lung cancer (NSCLC); however, long-term efficacy of these agents as monotherapies is limited and resistance often occurs (Tabernero, 2007; Domvri *et al*, 2013). In this context, preclinical models suggest that resistance to EGFR-targeted agents can be associated with increased VEGF expression, and combined EGFR/VEGR pathway blockade can abrogate this resistance (Naumov *et al*, 2009). The commonality and cross-talk between these two prominent signalling pathways provides biological rationale for combined EGFR/VEGF pathway-targeted therapy. This rationale was tested in a Phase III trial in NSCLC patients, wherein the combination of bevacizumab, an anti-VEGF monoclonal antibody, and the reversible EGFR tyrosine kinase inhibitor (TKI) erlotinib was compared with erlotinib monotherapy (Herbst *et al*, 2011). The combination appeared to improve objective response and progression-free survival (PFS) *vs* erlotinib alone, although without an overall survival benefit.

Afatinib is an oral, irreversible ErbB family blocker that inhibits signalling from all ErbB receptor homo- and heterodimers (Li *et al*, 2008; Solca *et al*, 2012). On the basis of significant improvements in PFS *vs* standard platinum-based chemotherapy in two pivotal Phase III studies (Sequist *et al*, 2013; Wu *et al*, 2014), afatinib (40 mg, once daily (QD)) was approved in the USA, EU and Japan for the treatment of patients with NSCLC harbouring distinct types of EGFR mutations (European Medicines Agency, 2013; Food and Drug Administration, 2013; Pharmaceuticals and Medical Devices Agency Japan, 2013). Nintedanib is an oral triple angiokinase inhibitor of VEGFR-1, 2 and 3, platelet-derived growth factor receptor (PDGFR) *α* and *β*, and fibroblast growth factor receptor (FGFR) 1, 2 and 3 (Hilberg *et al*, 2008). Nintedanib (200 mg, twice daily (BID)) is approved in the EU in combination with docetaxel for second-line treatment of patients with advanced NSCLC of adenocarcinoma histology (European Medicines Agency, 2015), and in the USA and EU for the treatment of patients with idiopathic pulmonary fibrosis (at a dose of 150 mg BID) (European Medicines Agency, 2014; Food and Drug Administration, 2014). The combination of afatinib and nintedanib has been evaluated in preclinical studies and early phase clinical trials in advanced solid tumours. In preclinical models of head and neck squamous cell carcinoma (HNSCC) and colorectal cancer, the combination of afatinib plus nintedanib demonstrated greater inhibition of cell proliferation and xenograft tumour growth compared with either single agent (Solca *et al*, 2007; Poindessous *et al*, 2011). In two Phase II studies investigating sequential combination of afatinib and nintedanib in patients with advanced colorectal cancer or castration-resistant prostate cancer, the combination treatment was associated with expected and manageable safety profiles but showed limited antitumour activity (Bouche *et al*, 2011; Molife *et al*, 2014). It was speculated that more intensive, continuous dosing may provide greater efficacy and would be feasible based on the manageable safety findings (Bouche *et al*, 2011).

On the basis of the aforementioned clinical studies, new dosing schedules were devised for afatinib, administered either continuously or intermittently, in combination with continuous nintedanib. This Phase I study, which evaluated the new dosing schedules in patients with advanced solid tumours, consisted of two parts: a dose-escalation phase and an expansion phase (ClinicalTrials.gov Identifier: NCT00998296; 1239.14). The dose-escalation phase was conducted to evaluate the safety and maximum tolerated dose (MTD) of the combination dosing schedules. The expansion phase investigated the safety and preliminary antitumour activity of the combination at the MTD in a concomitant, continuous treatment schedule in patients with NSCLC or pancreatic adenocarcinoma.

## Materials and methods

### Patients

Patients were ⩾18 years of age, with confirmed diagnosis of advanced solid tumours not amenable to established treatments, a life expectancy of ⩾3 months, and an Eastern Cooperative Oncology Group (ECOG) performance status of 0 or 1. Additional inclusion criteria included adequate organ function with no significant gastrointestinal tract or cardiovascular disease, pre-existing interstitial lung disease, active infective disease, or untreated or symptomatic brain metastases. Patients were excluded if they had received chemotherapy, immunotherapy, radiotherapy (small-field palliative radiotherapy was allowed), hormone therapy (except for breast or prostate cancer treatment), prior EGFR- or HER2-inhibiting drugs, an anti-angiogenic agent, or any other investigational drugs within 4 weeks prior to screening; had a history of haemorrhagic or thrombotic events within the last 6 months; or current Grade 1 or higher peripheral neuropathy unless due to trauma. No anticoagulation therapy, except low-dose heparin and/or heparin flush, was allowed.

The study was conducted in accordance with the Declaration of Helsinki, local laws and the International Conference on Harmonisation Good Clinical Practice Guideline, and approved by the relevant regulatory authority (Agence Nationale de Sécurité du Médicament et des Produits de Sante [ANSM], France) and an independent ethics committee (Comité de Protection des Personnes Ile de France X, Hôpital Robert Ballanger, France). All patients provided written informed consent for participation in the trial.

### Study design and treatments

This was a single-centre (France), open-label, Phase I study consisting of a dose-escalation phase and an expansion phase. The dose-escalation phase consisted of a modified 3+3 design with two schedules of afatinib in combination with nintedanib. Patients received 10, 20, 30 or 40 mg afatinib QD, following a continuous or intermittent schedule (every other week), in combination with continuous nintedanib (150 or 200 mg BID) for a 28-day treatment cycle. Dose-escalation started with 10 mg of afatinib QD administered continuously. If after 28 days fewer than two patients had experienced dose-limiting toxicities (DLTs), a new cohort at the next dose level was started. Each cohort consisted of at least three patients. Dose escalation of continuous afatinib continued until observation of DLTs in two or more patients during the first 28-day cycle; the same dose level was then explored for the intermittent afatinib schedule. Nintedanib was initially administered at a fixed dose of 200 mg BID; however, due to excess DLTs no MTD was determined. Following a protocol amendment, the nintedanib dose was reduced to 150 mg BID and a second dose-escalation phase was conducted starting with afatinib 30 mg QD.

In the expansion phase, treatment of two additional cohorts of patients with NSCLC or pancreatic adenocarcinoma was initiated at the MTD for concomitant afatinib plus nintedanib therapy determined in the dose-escalation phase. Treatment continued for as long as tolerable and no progressive disease (PD) was observed.

### Endpoints and assessments

The primary endpoint of the dose-escalation phase was to determine the MTD of the afatinib and nintedanib combination, defined as the highest doses of afatinib (continuous or intermittent) and nintedanib at which fewer than two of six treated patients experienced a DLT during the first 28-day treatment cycle. Exploratory secondary endpoints for the dose-escalation phase included safety, objective response according to Response Evaluation Criteria In Solid Tumours (RECIST) version 1.1, pharmacokinetic (PK) assessments and numbers of circulating tumour cells (CTCs). The primary endpoints of the expansion phase were to assess the safety and preliminary antitumour activity of concomitant afatinib plus nintedanib at the previously determined MTD in patients with NSCLC or pancreatic adenocarcinoma.

Safety was assessed by incidence and intensity of adverse events (AEs) graded according to the National Cancer Institute (NCI) Common Terminology Criteria for Adverse Events (CTCAE) version 3.0 (http://ctep.cancer.gov/protocolDevelopment/electronic_applications/ctc.htm). Recommendations for afatinib or nintedanib dose interruptions and reductions were provided to effectively manage specific treatment-related AEs. In the event of any treatment-related Grade ⩾3 AE, Grade ⩾2 diarrhoea persisting for ⩾2 consecutive days despite supportive care (i.e., treatment with antidiarrhoeal medication and hydration) or Grade ⩾3 diarrhoea despite supportive care, treatment with afatinib was interrupted until recovery to Grade ⩽1 or baseline (i.e., resolution to at least the patient's pretherapy value at study enrolment). In the event of treatment-related Grade ⩾3 aspartate aminotransferase (AST) and/or alanine aminotransferase (ALT) elevations or Grade ⩾2 elevation in conjunction with Grade ⩾1 bilirubin elevation, treatment with nintedanib was interrupted until recovery to baseline. Once recovered, patients could continue treatment with the assigned study medication following recommended dose reductions, depending on the starting dose. For patients receiving afatinib 30 or 40 mg QD, 10-mg dose reductions to a minimum of 20 mg QD were allowed; treatment was discontinued in case of further AEs at 20 mg QD. Afatinib 10 and 20 mg QD starting doses were not reduced but given intermittently; treatment was discontinued in case of further AEs. Nintedanib doses were reduced by 50-mg decrements to a minimum of 100 mg BID (from an initial dose of 200 mg BID) or 50 mg BID (from an initial dose of 150 mg BID); treatment was discontinued in case of further AEs.

Treatment-related AEs defined as DLTs per protocol included: uncomplicated Grade 4 neutropenia for >7 days; Grade 3/4 neutropenia of any duration associated with fever >38.5 °C; platelets <25 000 per μl or Grade 3 thrombocytopenia associated with bleeding requiring transfusion; Grade ⩾3 non-haematological toxicity (except nausea, vomiting or diarrhoea if managed with appropriate medical care, and alkaline phosphatase (ALP) <10 × the upper limit of normal (ULN) in patients with Grade 2 ALP (>2.5–5 × ULN) at baseline due to bone or liver metastases); Grade ⩾2 decrease in cardiac left ventricular function, worsening of renal function, or diarrhoea, vomiting or nausea persisting for ⩾7 days, despite supportive care; treatment-related liver toxicity except gamma glutamyltransferase elevation (AST/ALT/ALP >5 × ULN if total bilirubin ⩽1.5 × ULN or AST/ALT/ALP >2.5 × ULN if associated with total bilirubin >1.5 × ULN); Grade ⩾2 DLTs leading to an interruption of both drugs for ⩾14 consecutive days. For any patient with a DLT, treatment was stopped until the AE resolved to Grade ⩽1 or baseline. Once recovered, patients who had a documented clinical benefit (absence of disease progression) were eligible for further treatment with the assigned study medication following recommendations for dose reduction, as described above.

Tumour assessments by computed tomography or magnetic resonance imaging were performed at screening, every 6 weeks after starting study treatment until disease progression, and at the end-of-treatment visit. Tumour response was evaluated according to RECIST (version 1.1). Investigators assigned one of the following categories to each patient: complete response (CR), partial response (PR), stable disease (SD), PD or not evaluable. Disease control rate ((DCR) including CR, PR and SD) was also assessed.

During the dose-escalation phase, PK assessments were conducted in all patients who took at least one dose of trial medication and provided at least one blood sample following drug administration. Samples (5 ml of venous blood) were collected immediately before the morning administration of treatment on Days 1, 8, 15, 22, 28, 42 and 56. Afatinib and nintedanib drug concentrations were determined by validated high-performance liquid chromatography—tandem mass spectrometry.

For determination of CTCs, 7.5 ml blood samples were drawn in CellSave Blood Collection tubes (Veridex, Raritan, NJ, USA) at baseline and at Days 15, 30 and 60. Samples were analysed with CellSearch Circulating Tumour kit (Veridex, Raritan, NJ, USA) according to the manufacturer's instructions and reported as number of CTCs in 7.5 ml of blood. Patients were categorised as having unfavourable (⩾5 CTC/7.5 ml) or favourable (<5 CTC/7.5 ml) CTC counts, based on defined cut-offs from previous studies (Cristofanilli *et al*, 2004; de Bono *et al*, 2008).

### Statistical analyses

The number of patients required to determine the MTD in the dose-escalation phase ranged from 3 to 45, depending on the dosage reached. In the expansion phase, a maximum of 12 patients were to be recruited for the pancreatic adenocarcinoma cohort, and a maximum of 18 patients were to be recruited for the NSCLC cohort. Assuming a 5% drop out rate, the estimated number of patients required for the study was up to 79.

All analyses are descriptive and exploratory. Data from the two afatinib dosing schedules in the dose-escalation phase were analysed separately. The treated analysis set included all patients who received at least one dose of afatinib or nintedanib during the study period. The MTD analysis set included all patients eligible for determination of MTD in the opinion of the investigator (reviewed by the Clinical Trial Monitor).

## Results

### Patients

A total of 45 patients were enroled in the dose-escalation phase from October 2009 to January 2012 (Table 1). The majority of patients had an ECOG performance status of 1 (69%) and had received at least four lines of prior anticancer therapy (73%). The most common cancer types were colorectal, NSCLC, ovary and breast.

Following the dose-escalation phase, 25 patients with NSCLC (*n*=18) or pancreatic adenocarcinoma (*n*=7) were enroled in the expansion phase (Table 1). The majority of patients with NSCLC were female (61%), whereas the majority of patients with pancreatic adenocarcinoma were male (86%). Among all patients in the expansion phase, 44% received at least four lines of prior anticancer therapy.

### DLTs and determination of MTD in the dose-escalation phase

Dose escalation up to 40 mg afatinib QD was achieved for both intermittent and continuous schedules. Overall, there were 12 DLTs reported in seven patients receiving afatinib in a continuous schedule and five DLTs in four patients receiving afatinib in an intermittent schedule; the frequency and intensity of DLTs observed in each dose cohort are detailed in Table 2. The most frequent DLT was diarrhoea (five patients (11%)) followed by increased ALT, increased blood creatinine, dehydration, hepatocellular injury and acute renal failure (two patients (4%) each); DLTs of increased AST and hepatotoxicity occurred in one patient each. The maximum CTCAE grade reported for DLTs was Grade 3 for 10 patients and Grade 4 for one patient (hepatotoxicity in the nintedanib 200 mg BID plus afatinib 40 mg QD intermittent cohort). Two dose cohorts achieved six evaluable patients with no DLTs, therefore two MTDs were determined: nintedanib 150 mg BID plus afatinib 30 mg QD continuously, and nintedanib 150 mg BID plus afatinib 40 mg QD every other week. The continuous afatinib 30 mg QD plus nintedanib 150 mg BID schedule was chosen for further evaluation in the expansion phase of the trial, based on the findings from two Phase II studies demonstrating a lack of antitumour activity with intermittent afatinib plus nintedanib scheduling (Bouche *et al*, 2011; Molife *et al*, 2014).

### Treatment duration

In the dose-escalation phase, median (range) exposure to afatinib and nintedanib in the continuous cohorts was 62.5 (7–239) days. In the intermittent cohorts, median exposure to afatinib and nintedanib was 53.0 (4–160) days and 60.0 (11–167) days, respectively. Eight patients discontinued treatment before the end of the first 28-day treatment cycle; two (4%) for progressive disease, five (11%) for DLT or dose-reducing toxicity, and one (2%) for Grade 3 diarrhoea.

In the total expansion phase population, median (range) exposure to continuous afatinib and nintedanib was 43.0 (2–307) and 44.0 (2–308) days, respectively. Median exposure to afatinib and nintedanib was longer in patients with NSCLC (78.5 (14–307) and 84.0 (7–308) days, respectively) than in patients with pancreatic adenocarcinoma (43.0 (2–43) and 42.0 (2–44) days, respectively). The most common reason for study discontinuation was progressive disease (76% of total patients; 78% NSCLC and 71% pancreatic adenocarcinoma patients), followed by AEs (12% one NSCLC and two pancreatic adenocarcinoma patients), patient refusal to continue trial medication (two NSCLC patients) and death (one NSCLC patient).

### Overall safety

In the dose-escalation phase, all patients experienced at least one AE, with treatment-related AEs occurring in 44 (98%) patients (Table 3). Most treatment-related AEs were Grade ⩽3; no Grade 5 AEs were reported. Diarrhoea (98%), asthenia (64%), nausea (62%) and vomiting (60%) were the most frequently occurring treatment-related AEs. Eight patients (18%) had AEs that led to discontinuation of afatinib and nintedanib, and two patients had AEs that led to discontinuation of afatinib only (one receiving intermittent afatinib 40 mg QD and nintedanib 150 mg BID and one receiving intermittent afatinib 40 mg QD and nintedanib 200 mg BID). Serious AEs (SAEs) occurred in 26 patients (17 on continuous afatinib and 9 on intermittent afatinib). Among these patients, 15 (58%) had at least one SAE that was considered to be treatment-related. There were three deaths, all due to AEs that occurred post treatment (within 28 days following the last treatment administration). In the cohorts receiving the determined MTD, SAEs occurred in 3/6 patients receiving continuous afatinib 30 mg QD plus nintedanib 150 mg BID (no patient discontinued treatment due to AEs), and 3/7 patients receiving intermittent afatinib 40 mg QD plus nintedanib 150 mg BID (one patient discontinued afatinib due to AEs: dehydration, decreased appetite, diarrhoea and vomiting).

In the expansion phase, all patients experienced at least one AE, with treatment-related AEs occurring in 24 (96%) patients (Supplementary Table 1). All treatment-related AEs were Grade ⩽3, with diarrhoea (88%), rash (56%), asthenia (52%), decreased appetite (48%) and nausea (48%) occurring most frequently. AEs led to discontinuation of afatinib in three NSCLC patients, nintedanib in two NSCLC patients, and both afatinib and nintedanib in one NSCLC patient and two pancreatic adenocarcinoma patients. SAEs were reported in 10 (40%) patients, with similar incidences in NSCLC (39%) and pancreatic adenocarcinoma (43%) patients. There were two deaths due to AEs in patients with NSCLC, neither of which were considered treatment-related; one occurred while on treatment and one post treatment (within 28 days following the last treatment administration).

### Antitumour activity

**Figure 1:**
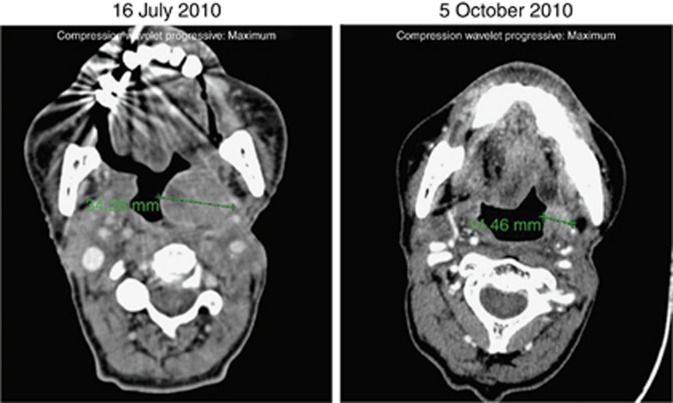
**Partial response (−58% change in tumour lesions^a^) in a patient with squamous cell carcinoma of the epiglottis receiving intermittent afatinib 40 mg QD plus continuous 150 mg nintedanib BID.** ^a^Change in measurement from 34.26 mm (16 July 2010) to 14.46 mm (5 October 2010). Abbreviations: BID= twice daily; QD= once daily.

**Figure 2:**
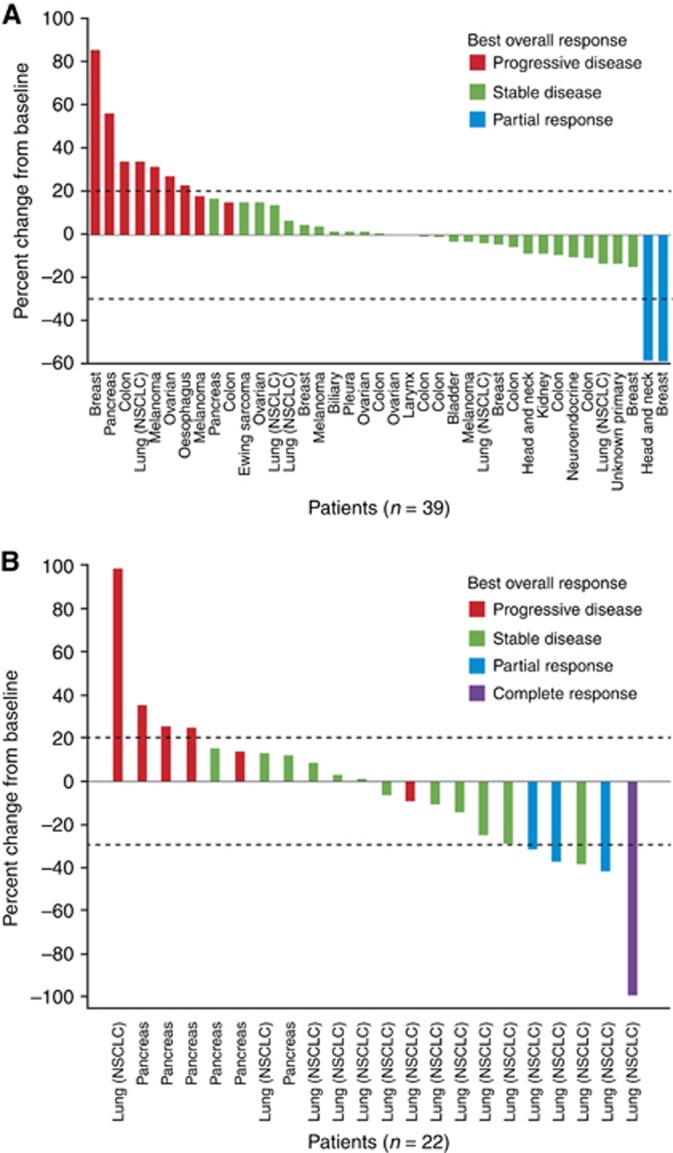
Per cent change from baseline and best overall response in the dose-escalation phase^a^ (**A**) and expansion phase (**B**). ^a^Includes both target and non-target lesions, and the occurrence of new lesions. Abbreviation: NSCLC=non-small cell lung cancer.

Of 40 patients in the dose-escalation phase evaluable for tumour response, two PRs were observed; one in a triple-negative breast cancer patient and the other in a HNSCC patient. Both patients received intermittent afatinib 40 mg QD plus continuous nintedanib 200 mg BID (Figure 1). SD was reported as the best overall response in 60% (27/45) of patients (Figure 2A), lasting >12 weeks in eight patients. The overall DCR was 64% (29/45).

In the expansion phase, one CR and three PRs were observed in NSCLC patients treated with concomitant, continuous afatinib 30 mg QD plus nintedanib 150 mg BID (Table 4). The patient with CR was a 66-year-old male with EGFR mutation-positive NSCLC who had been heavily pretreated with five previous lines of therapy (including platinum- and taxane-based regimens with or without bevacizumab, and gefitinib). Of the three patients with PRs, all were heavily pretreated (at least two previous lines of therapy), failing prior combination chemotherapy and EGFR-TKI therapy, and one patient had EGFR mutation-positive disease. SD was observed in 61% (11/18) of NSCLC patients (lasting >12 weeks in eight patients) and 29% (2/7) of pancreatic adenocarcinoma patients; the overall DCR in NSCLC patients was 83% (15/18). Percent changes from baseline in target lesions for patients treated in the expansion phase are displayed in Figure 2B.

### Pharmacokinetics

Mean trough plasma concentrations of afatinib during continuous dosing with afatinib 30 mg QD plus nintedanib 150 mg BID were similar to those observed in previous trials of afatinib 30 mg QD as monotherapy (Supplementary Figure 1). Similarly, mean trough plasma concentrations of nintedanib during dosing with nintedanib 150 mg BID plus continuous afatinib 30 mg QD were similar to those in previous nintedanib monotherapy trials. No significant drug–drug interactions between either dosing schedule of afatinib and nintedanib were observed.

### Analyses of CTCs

Exploratory analysis of CTCs was conducted in 40 patients treated in the dose-escalation phase who had epithelial tumours (Supplementary Table 2). Of 35 patients evaluable for tumour response, the DCR was 76% (19/25) among those with a favourable CTC count (<5 CTC/7.5 ml) at baseline, and 24% (6/25) among patients with an unfavourable CTC count at baseline. The two patients with PR had detectable CTCs at baseline that were undetectable at Day 60.

## Discussion

In this Phase I study in patients with advanced solid tumours, MTDs were established for both continuous and intermittent schedules of afatinib in combination with nintedanib in the dose-escalation phase: afatinib 40 mg QD intermittently (every other week) plus nintedanib 150 mg BID, or afatinib 30 mg QD continuously plus nintedanib 150 mg BID. On the basis of the results from two Phase II studies demonstrating a lack of antitumour activity with intermittent schedules of combined afatinib and nintedanib in patients with hormone-refractory prostate cancer (Molife *et al*, 2014) and advanced colorectal cancer (Bouche *et al*, 2011), the continuous afatinib 30 mg QD plus nintedanib 150 mg BID schedule was chosen for further evaluation in the expansion phase of the study.

The safety profile of afatinib and nintedanib combination therapy in both the dose-escalation and expansion phases was generally manageable and consistent with previous observations for the individual agents, with no new safety concerns identified (Mross *et al*, 2010; Yap *et al*, 2010; Doebele *et al*, 2012). In the dose-escalation phase, the most common DLTs were diarrhoea and transaminase elevations; these findings are consistent with previous combination studies of afatinib and nintedanib cancer (Bouche *et al*, 2011; Molife *et al*, 2014). In both phases of the study, the most common AEs were gastrointestinal, mainly diarrhoea. The majority of these events were Grade ⩽3, and few study discontinuations due to diarrhoea were observed (one at the MTD of the intermittent schedule in the dose-escalation phase and three in the expansion phase); this was likely a result of early intervention and robust diarrhoea management in this study. In this context, another Phase I study of afatinib in combination with nintedanib in patients with advanced solid tumours reported a lower MTD of continuous afatinib 10 mg QD plus nintedanib 200 mg BID, which the authors attributed to excessive dose-limiting diarrhoea in the absence of robust management of this AE (Gordon *et al*, 2015). Proactive management of diarrhoea is known to be crucial for achieving optimal clinical benefit with afatinib monotherapy (Yang *et al*, 2013).

Mean trough plasma concentrations of afatinib and nintedanib were similar to those observed in previous combination therapy and monotherapy studies (Mross *et al*, 2010; Yap *et al*, 2010; Bousquet *et al*, 2011; Doebele *et al*, 2012; Gordon *et al*, 2013; Marshall *et al*, 2013a, 2013b). Overall, PK assessments suggest no drug–drug interactions between afatinib and nintedanib.

In this heavily pretreated population, afatinib plus nintedanib combination therapy demonstrated some antitumour activity in both the dose-escalation and expansion phases of the study. In the dose-escalation phase, one patient with triple-negative breast cancer and one with HNSCC (both on the MTD of the intermittent schedule) had a PR, and 60% of patients had SD. In the expansion phase, in patients treated with concomitant, continuous afatinib 30 mg QD plus nintedanib 150 mg BID, one CR and three PRs were observed in patients with NSCLC (with SD observed in 61% of patients); two patients with pancreatic adenocarcinoma achieved SD. These results are encouraging compared with the lack of antitumour activity observed in previous Phase II studies in patients with advanced colorectal cancer and hormone-refractory prostate cancer (Bouche *et al*, 2011; Molife *et al*, 2014). The clinical activity observed in this study suggests that the intermittent combination dosing explored in the previous trials was suboptimal, and a continuous dosing regimen may be more suitable for further development of the afatinib and nintedanib combination.

Several studies, including patients with metastatic colorectal cancer, breast cancer and castrate-resistant prostate cancer, have demonstrated an association between patient CTC counts and clinical outcomes (Cristofanilli *et al*, 2004; Cohen *et al*, 2008; de Bono *et al*, 2008). In an exploratory analysis of CTCs in this study, there was a higher proportion of patients with a favourable CTC count (<5 CTC/7.5 ml) among those with PR or SD compared to those with PD. These results should be interpreted with a degree of caution due to the small number of patients included in this analysis, and warrant further analysis in a larger population.

In summary, the combination of either afatinib 40 mg QD every other week plus nintedanib 150 mg BID, or continuous afatinib 30 mg QD plus nintedanib 150 mg BID was associated with a manageable AE profile and showed evidence of antitumour activity in this heavily pretreated patient population. However, appropriate and proactive management of diarrhoea will be fundamental to improving patient quality of life and treatment outcomes. The clinical activity of continuous afatinib 30 mg QD plus nintedanib 150 mg BID was further supported in expanded analyses of patients with NSCLC or pancreatic adenocarcinoma. Taken together, these findings confirm the feasibility of dual EGFR/VEGFR blockade via concomitant, continuous afatinib plus nintedanib therapy in patients with advanced solid tumours. Further investigation of this treatment regimen in future trials is warranted if the expected benefits outweigh the potential risks.

## Figures and Tables

**Table 1:** Summary of baseline demographic and disease characteristics

**Characteristic**	**Dose-escalation phase (*****n*****=45)**	**Expansion phase (*****n*****=25)**
Median age, years (range)	56 (37–73)	58 (37–72)
**Gender, *n* (%)**		
Male	26 (58)	13 (52)
**ECOG PS,** ***n*** **(%)**		
0	14 (31)	11 (44)
1	31 (69)	14 (56)
**Tumour type,** ***n*** **(%)**		
Colorectal	9 (20)	—
NSCLC	6 (13)	18 (72)
Ovary	6 (13)	—
Breast	5 (11)	—
Melanoma	4 (9)	—
HNSCC	3 (7)	—
Pancreas	2 (4)	7 (28)
Endocrine	2 (4)	—
Other^a^	8 (18)	—
**Lines of previous systemic anticancer therapy**^b^		
1	3 (7)	2 (8)
2	4 (9)	4 (16)
3	4 (9)	8 (32)
At least 4	33 (73)	11 (44)
**Afatinib administration,** ***n*** **(%)**		
Continuous	26 (58)	25 (100)
Intermittent	19 (42)	—

Abbreviations: ECOG PS=Eastern Cooperative Oncology Group performance status; HNSCC=head and neck squamous cell carcinoma; NSCLC=non-small cell lung cancer.

a Other tumour types include one patient each with soft tissue sarcoma, cancer of unknown primary, biliary tree, bladder, pleura, thyroid, oesophagus and kidney.

b One patient in the dose-escalation phase did not receive previous systemic therapy.

**Table 2:** Summary of DLTs occurring during the first treatment cycle (28 days)

	**Cohort 1**	**Cohort 2**	**Cohort 3**	**Cohort 4**	**Cohort 5**	**Cohort 6**	**Cohort 7**	**Cohort 8**	**Cohort 9**	**All patients**
**Afatinib dose mg QD**	**10 (C)**	**20** **(C)**	**30 (C)**	**40 (C)**	**30 (I)**	**40 (I)**	**40 (C)**	**40 (I)**	**30 (C)**	
**Nintedanib dose mg BID**	**200**	**200**	**200**	**200**	**200**	**200**	**150**	**150**	**150**	
Patients treated, *n*	3	3	8	3	6	6	3	7	6	45
Patients evaluable for MTD, *n*	3	3	6^a^	3	6	6	3	6^b^	6	42
Patients with DLT, *n* (%)			2 (25)	3 (100)	2 (33.3)	2 (33.3)	2 (66.7)			11 (24.4)
**Dose-limiting toxicities occurring during the first 28-day treatment cycle, (G)**										
			Diarrhoea (G3)	ALT increased (G3)	Hepatocellular injury (G3)	Dehydration (G3)	Acute renal failure (G3)			
	0	0	ALT increased, AST increased and diarrhoea (all G3)	Diarrhoea (G3)	Diarrhoea and blood creatinine increased (both G3)	Hepatotoxicity (G4)	Diarrhoea, dehydration and acute renal failure (all G3)	0	0	
				Blood creatinine increased (G2) and hepatocellular injury (G3)						

Abbreviations: ALT=alanine aminotransferase; AST=aspartate aminotransferase; BID=twice daily; C=continuous; DLT=dose-limiting toxicity; I=intermittent; G=Grade; MTD=maximum tolerated dose; QD=once daily.

a Poor compliance for one patient and treatment exposure for less than 21 days due to consent withdrawal for another patient.

b Premature discontinuation due to clinical progression for one patient.

**Table 3:** Treatment-related AEs by NCI-CTCAE grade^a^ occurring in >10% of patients in the dose-escalation phase

**AE,** ***n*** **(%)**	**Grade 1**	**Grade 2**	**Grade 3**	**Grade 4**	**Total (*****n*****=45)**
Any AE	2 (4)	13 (29)	26 (58)	3 (7)	44 (98)
Diarrhoea	8 (18)	17 (38)	19 (42)	0 (0)	44 (98)
Asthenia	11 (24)	15 (33)	3 (7)	0 (0)	29 (64)
Nausea	22 (49)	6 (13)	0 (0)	0 (0)	28 (62)
Vomiting	14 (31)	13 (29)	0 (0)	0 (0)	27 (60)
Decreased appetite	15 (33)	7 (16)	4 (9)	0 (0)	26 (58)
Folliculitis	19 (42)	4 (9)	0 (0)	0 (0)	23 (51)
Rhinitis	18 (40)	1 (2)	0 (0)	0 (0)	19 (42)
Epistaxis	18 (40)	0 (0)	0 (0)	0 (0)	18 (40)
Dry skin	17 (38)	0 (0)	0 (0)	0 (0)	17 (38)
ALT increased	12 (27)	2 (4)	3 (7)	0 (0)	17 (38)
Dry mouth	13 (29)	0 (0)	0 (0)	0 (0)	13 (29)
AST increased	9 (20)	2 (4)	2 (4)	0 (0)	13 (29)
Hypokalaemia	7 (16)	0 (0)	5 (11)	1 (2)	13 (29)
Mucosal inflammation	6 (13)	5 (11)	0 (0)	0 (0)	11 (24)
Rash	11 (24)	0 (0)	0 (0)	0 (0)	11 (24)
Hepatocellular injury	4 (9)	4 (9)	2 (4)	0 (0)	10 (22)
Skin fissures	10 (22)	0 (0)	0 (0)	0 (0)	10 (22)
Muscle spasms	8 (18)	1 (2)	0 (0)	0 (0)	9 (20)
Dehydration	0 (0)	3 (7)	5 (11)	0 (0)	8 (18)
Abdominal pain	6 (13)	0 (0)	0 (0)	0 (0)	6 (13)
Aphthous stomatitis	3 (7)	3 (7)	0 (0)	0 (0)	6 (13)
Dysgeusia	6 (13)	0 (0)	0 (0)	0 (0)	6 (13)
Onychoclasis	5 (11)	0 (0)	0 (0)	0 (0)	5 (11)
Rhinorrhoea	5 (11)	0 (0)	0 (0)	0 (0)	5 (11)

Abbreviations: AE=adverse event; ALT=alanine aminotransferase; AST=aspartate aminotransferase; NCI-CTCAE=National Cancer Institute Common Terminology Criteria for Adverse Events.

a There were no Grade 5 treatment-related AEs.

**Table 4:** Best overall response in the expansion phase

**Best overall response,** ***n*** **(%)**	**NSCLC (*****n*****=18)**	**Pancreatic adenocarcinoma (*****n*****=7)**	**Total (*****n*****=25)**
Objective response^a^	4 (22)	0 (0)	4 (16)
Disease control^b^	15 (83)	2 (29)	17 (68)
Complete response	1 (6)	0 (0)	1 (4)
Partial response	3 (17)	0 (0)	3 (12)
Stable disease	11 (61)	2 (29)	13 (52)
Progressive disease	2 (11)	4 (57)	6 (24)
Not evaluated^c^	1 (6)	1 (14)	2 (8)

Abbreviation: NSCLC=non-small cell lung cancer.

a Includes complete and partial response.

b Includes objective response and stable disease.

c No assessment was performed.

## References

[bib1] Bouche O, Maindrault-Goebel F, Ducreux M, Lledo G, Andre T, Stopfer P, Amellal N, Merger M, De GA (2011) Phase II trial of weekly alternating sequential BIBF 1120 and afatinib for advanced colorectal cancer. Anticancer Res 31: 2271–2281. 21737652

[bib2] Bousquet G, Alexandre J, Le TC, Goldwasser F, Faivre S, de Mont-Serrat H, Kaiser R, Misset JL, Raymond E (2011) Phase I study of BIBF 1120 with docetaxel and prednisone in metastatic chemo-naive hormone-refractory prostate cancer patients. Br J Cancer 105: 1640–1645. 22027711 10.1038/bjc.2011.440 PMC3242598

[bib3] Cohen SJ, Punt CJ, Iannotti N, Saidman BH, Sabbath KD, Gabrail NY, Picus J, Morse M, Mitchell E, Miller MC, Doyle GV, Tissing H, Terstappen LW, Meropol NJ (2008) Relationship of circulating tumor cells to tumor response, progression-free survival, and overall survival in patients with metastatic colorectal cancer. J Clin Oncol 26: 3213–3221. 18591556 10.1200/JCO.2007.15.8923

[bib4] Cristofanilli M, Budd GT, Ellis MJ, Stopeck A, Matera J, Miller MC, Reuben JM, Doyle GV, Allard WJ, Terstappen LW, Hayes DF (2004) Circulating tumor cells, disease progression, and survival in metastatic breast cancer. N Engl J Med 351: 781–791. 15317891 10.1056/NEJMoa040766

[bib5] de Bono JS, Scher HI, Montgomery RB, Parker C, Miller MC, Tissing H, Doyle GV, Terstappen LW, Pienta KJ, Raghavan D (2008) Circulating tumor cells predict survival benefit from treatment in metastatic castration-resistant prostate cancer. Clin Cancer Res 14: 6302–6309. 18829513 10.1158/1078-0432.CCR-08-0872

[bib6] Doebele RC, Conkling P, Traynor AM, Otterson GA, Zhao Y, Wind S, Stopfer P, Kaiser R, Camidge DR (2012) A phase I, open-label dose-escalation study of continuous treatment with BIBF 1120 in combination with paclitaxel and carboplatin as first-line treatment in patients with advanced non-small-cell lung cancer. Ann Oncol 23: 2094–2102. 22345119 10.1093/annonc/mdr596 PMC4141207

[bib7] Domvri K, Zarogoulidis P, Darwiche K, Browning RF, Li Q, Turner JF, Kioumis I, Spyratos D, Porpodis K, Papaiwannou A, Tsiouda T, Freitag L, Zarogoulidis K (2013) Molecular targeted drugs and biomarkers in NSCLC, the evolving role of individualized therapy. J Cancer 4: 736–754. 24312144 10.7150/jca.7734 PMC3842443

[bib8] European Medicines Agency (2013) Afatinib—European Public Assessment Report (summary). Available at http://www.ema.europa.eu/ema/index.jsp?curl=pages/medicines/human/medicines/002280/human_med_001698.jsp&mid=WC0b01ac058001d124 (accessed on 31 August 2015).

[bib9] European Medicines Agency (2014) OFEV—European Public Assessment Report (summary). Available at http://www.ema.europa.eu/docs/en_GB/document_library/EPAR_-_Summary_for_the_public/human/003821/WC500182477.pdf (Accessed on 31 August 2015).

[bib10] European Medicines Agency (2015) Vargatef—European Public Assessment Report (summary). Available at http://www.ema.europa.eu/docs/en_GB/document_library/EPAR_-_Summary_for_the_public/human/002569/WC500179973.pdf (accessed on 31 August 2015).

[bib11] Food and Drug Administration (2013) Gilotrif prescribing information. Available at http://www.accessdata.fda.gov/drugsatfda_docs/label/2013/201292s000lbl.pdf (accessed on 31 August 2015).

[bib12] Food and Drug Administration (2014) OFEV prescribing information. Available at http://www.accessdata.fda.gov/drugsatfda_docs/label/2014/205832s000lbl.pdf (accessed on 31 August 2015).

[bib13] Goel HL, Mercurio AM (2013) VEGF targets the tumour cell. Nat Rev Cancer 13: 871–882. 24263190 10.1038/nrc3627 PMC4011842

[bib14] Gordon MS, Mendelson DS, Gross M, Uttenreuther-Fischer M, Ould-Kaci M, Zhao Y, Stopfer P, Agus DB (2013) A phase I, open-label, dose-escalation study of continuous once-daily oral treatment with afatinib in patients with advanced solid tumors. Invest New Drugs 31: 409–416. 23242861 10.1007/s10637-012-9904-9 PMC3589633

[bib15] Gordon MS, Springett GM, Su YB, Ould-Kaci M, Wind S, Zhao Y, LoRusso PM (2015) A phase I dose-escalation study of afatinib combined with nintedanib in patients with advanced solid tumors. Future Oncol 11: 1479–1491. 25963426 10.2217/fon.15.50

[bib16] Herbst RS, Ansari R, Bustin F, Flynn P, Hart L, Otterson GA, Vlahovic G, Soh CH, O'Connor P, Hainsworth J (2011) Efficacy of bevacizumab plus erlotinib *versus* erlotinib alone in advanced non-small-cell lung cancer after failure of standard first-line chemotherapy (BeTa): a double-blind, placebo-controlled, phase 3 trial. Lancet 377: 1846–1854. 21621716 10.1016/S0140-6736(11)60545-X PMC4134127

[bib17] Hilberg F, Roth GJ, Krssak M, Kautschitsch S, Sommergruber W, Tontsch-Grunt U, Garin-Chesa P, Bader G, Zoephel A, Quant J, Heckel A, Rettig WJ (2008) BIBF 1120: triple angiokinase inhibitor with sustained receptor blockade and good antitumor efficacy. Cancer Res 68: 4774–4782. 18559524 10.1158/0008-5472.CAN-07-6307

[bib18] Larsen AK, Ouaret D, El OK, Petitprez A (2011) Targeting EGFR and VEGF(R) pathway cross-talk in tumor survival and angiogenesis. Pharmacol Ther 131: 80–90. 21439312 10.1016/j.pharmthera.2011.03.012

[bib19] Li D, Ambrogio L, Shimamura T, Kubo S, Takahashi M, Chirieac LR, Padera RF, Shapiro GI, Baum A, Himmelsbach F, Rettig WJ, Meyerson M, Solca F, Greulich H, Wong KK (2008) BIBW2992, an irreversible EGFR/HER2 inhibitor highly effective in preclinical lung cancer models. Oncogene 27: 4702–4711. 18408761 10.1038/onc.2008.109 PMC2748240

[bib20] Marshall J, Hwang J, Eskens FA, Burger H, Malik S, Uttenreuther-Fischer M, Stopfer P, Ould-Kaci M, Cohen RB, Lewis NL (2013a) A Phase I, open-label, dose escalation study of afatinib, in a 3-week-on/1-week-off schedule in patients with advanced solid tumors. Invest New Drugs 31: 399–408. 23161335 10.1007/s10637-012-9890-y PMC3589659

[bib21] Marshall J, Shapiro GI, Uttenreuther-Fischer M, Ould-Kaci M, Stopfer P, Gordon MS (2013b) Phase I dose-escalation study of afatinib, an ErbB family blocker, plus docetaxel in patients with advanced cancer. Future Oncol 9: 271–281. 23414476 10.2217/fon.12.195

[bib22] Molife LR, Omlin A, Jones RJ, Karavasilis V, Bloomfield D, Lumsden G, Fong PC, Olmos D, O'Sullivan JM, Pedley I, Hickish T, Jenkins P, Thompson E, Oommen N, Wheatley D, Heath C, Temple G, Pelling K, de Bono JS (2014) Randomized Phase II trial of nintedanib, afatinib and sequential combination in castration-resistant prostate cancer. Future Oncol 10: 219–231. 24490608 10.2217/fon.13.250

[bib23] Mross K, Stefanic M, Gmehling D, Frost A, Baas F, Unger C, Strecker R, Henning J, Gaschler-Markefski B, Stopfer P, de Rossi L, Kaiser R (2010) Phase I study of the angiogenesis inhibitor BIBF 1120 in patients with advanced solid tumors. Clin Cancer Res 16: 311–319. 20028771 10.1158/1078-0432.CCR-09-0694

[bib24] Naumov GN, Nilsson MB, Cascone T, Briggs A, Straume O, Akslen LA, Lifshits E, Byers LA, Xu L, Wu HK, Janne P, Kobayashi S, Halmos B, Tenen D, Tang XM, Engelman J, Yeap B, Folkman J, Johnson BE, Heymach JV (2009) Combined vascular endothelial growth factor receptor and epidermal growth factor receptor (EGFR) blockade inhibits tumor growth in xenograft models of EGFR inhibitor resistance. Clin Cancer Res 15: 3484–3494. 19447865 10.1158/1078-0432.CCR-08-2904 PMC2893040

[bib25] Pharmaceuticals and Medical Devices Agency Japan (2013) New drugs approved in FY 2013. Available at http://www.pmda.go.jp/english/service/pdf/list/NewdrugsFY2013.pdf (accessed on 31 August 2015).

[bib26] Poindessous V, Ouaret D, El OK, Battistella A, Megalophonos VF, Kamsu-Kom N, Petitprez A, Escargueil AE, Boudou P, Dumont S, Cervera P, Flejou JF, Andre T, Tournigand C, Chibaudel B, De GA, Larsen AK (2011) EGFR- and VEGF(R)-targeted small molecules show synergistic activity in colorectal cancer models refractory to combinations of monoclonal antibodies. Clin Cancer Res 17: 6522–6530. 21880790 10.1158/1078-0432.CCR-11-1607

[bib27] Sequist LV, Yang JC, Yamamoto N, O'Byrne K, Hirsh V, Mok T, Geater SL, Orlov S, Tsai CM, Boyer M, Su WC, Bennouna J, Kato T, Gorbunova V, Lee KH, Shah R, Massey D, Zazulina V, Shahidi M, Schuler M (2013) Phase III study of afatinib or cisplatin plus pemetrexed in patients with metastatic lung adenocarcinoma with EGFR mutations. J Clin Oncol 31: 3327–3334. 23816960 10.1200/JCO.2012.44.2806

[bib28] Solca F, Baum A, Krause M, Baumann M, Wong K, Greulich H, Guenther A (2007) Efficacy of BIBW 2992, a potent irreversible inhibitor of EGFR and HER2, in models of head and neck cancer. Eur J Cancer Suppl 5: 326–327.

[bib29] Solca F, Dahl G, Zoephel A, Bader G, Sanderson M, Klein C, Kraemer O, Himmelsbach F, Haaksma E, Adolf GR (2012) Target binding properties and cellular activity of afatinib (BIBW 2992), an irreversible ErbB family blocker. J Pharmacol Exp Ther 343: 342–350. 22888144 10.1124/jpet.112.197756

[bib30] Tabernero J (2007) The role of VEGF and EGFR inhibition: implications for combining anti-VEGF and anti-EGFR agents. Mol Cancer Res 5: 203–220. 17374728 10.1158/1541-7786.MCR-06-0404

[bib31] Wu YL, Zhou C, Hu CP, Feng J, Lu S, Huang Y, Li W, Hou M, Shi JH, Lee KY, Xu CR, Massey D, Kim M, Shi Y, Geater SL (2014) Afatinib *versus* cisplatin plus gemcitabine for first-line treatment of Asian patients with advanced non-small-cell lung cancer harbouring EGFR mutations (LUX-Lung 6): an open-label, randomised phase 3 trial. Lancet Oncol 15: 213–222. 24439929 10.1016/S1470-2045(13)70604-1

[bib32] Yang JC, Reguart N, Barinoff J, Kohler J, Uttenreuther-Fischer M, Stammberger U, O'Brien D, Wolf J, Cohen EE (2013) Diarrhea associated with afatinib: an oral ErbB family blocker. Expert Rev Anticancer Ther 13: 729–736. 23506556 10.1586/era.13.31

[bib33] Yap TA, Vidal L, Adam J, Stephens P, Spicer J, Shaw H, Ang J, Temple G, Bell S, Shahidi M, Uttenreuther-Fischer M, Stopfer P, Futreal A, Calvert H, de Bono JS, Plummer R (2010) Phase I trial of the irreversible EGFR and HER2 kinase inhibitor BIBW 2992 in patients with advanced solid tumors. J Clin Oncol 28: 3965–3972. 20679611 10.1200/JCO.2009.26.7278

[bib34] Yarden Y, Pines G (2012) The ERBB network: at last, cancer therapy meets systems biology. Nat Rev Cancer 12: 553–563. 22785351 10.1038/nrc3309

