# Supplementary material for: Phase I study of afatinib combined with nintedanib in patients with advanced solid tumours
Source: Br J Cancer. 2015 Oct 29;113(10):1413–20. doi: 10.1038/bjc.2015.374 (PMC4815889; doi:10.1038/bjc.2015.374)
Supplement: Supplementary Figure 1 [file bjc2015374x1.pdf]

**Supplementary Figure 1.** Individual and geometric mean trough plasma concentrations of afatinib and nintedanib (MTD cohorts) during combination therapy compared with values observed in monotherapy studies.

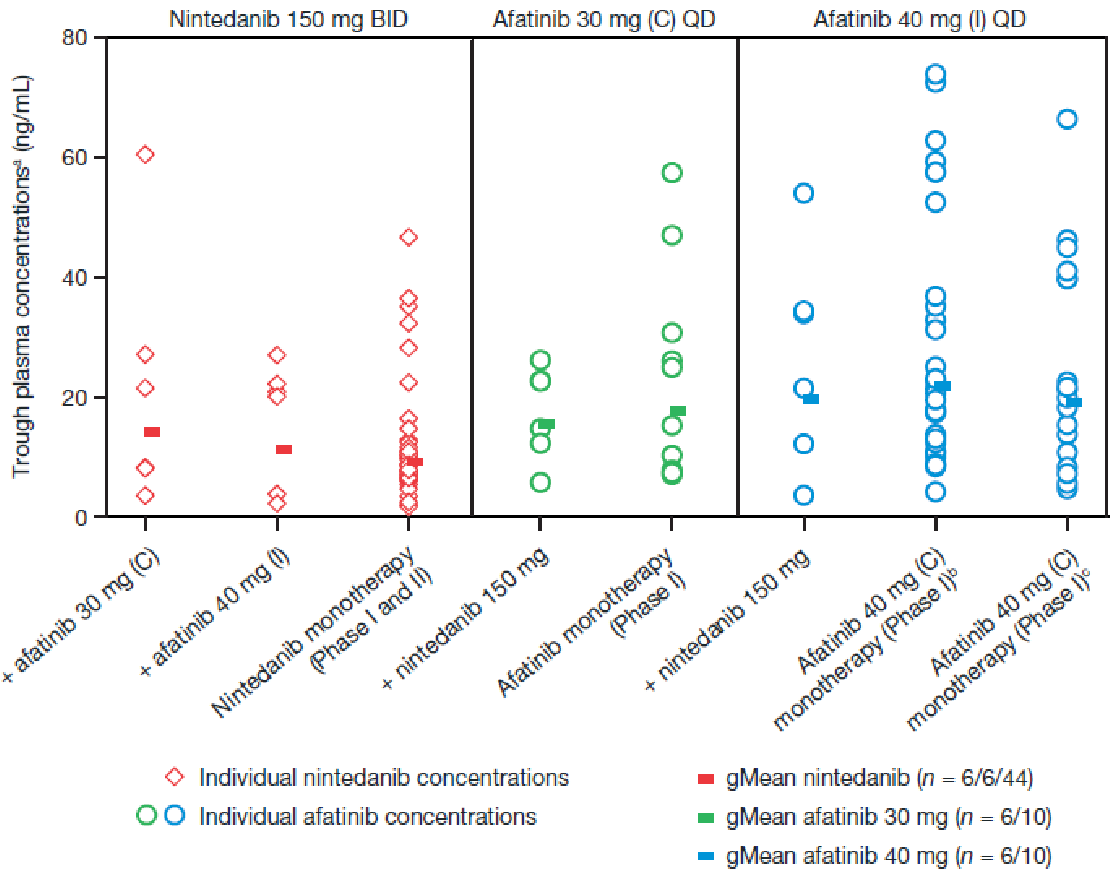


Abbreviations: BID=twice daily; C=continuous; gMean=geometric mean; I=intermittent; MTD=maximum tolerated dose; QD=once daily.

[a]At 336 hours (Day 15).

[b]From meta-analysis of afatinib in Phase I studies.

[c]From afatinib monotherapy arm in another Phase I study analysing the nintedanib/afatinib concentration.
