# Supplementary material for: Phase I study of afatinib combined with nintedanib in patients with advanced solid tumours
Source: Br J Cancer. 2015 Oct 29;113(10):1413–20. doi: 10.1038/bjc.2015.374 (PMC4815889; doi:10.1038/bjc.2015.374)
Supplement: Supplementary Table 1 [file bjc2015374x2.pdf]

Supplementary Table 1. Treatment-related AEs by NCI-CTCAE grade[a] occurring in >10% of patients in the expansion phase

**AE, *n* (%)**	**Grade 1**	**Grade 2**	**Grade 3**	**Total (*n* = 25)**
Any AE	2 (8)	11 (44)	11 (44)	24 (96)
Diarrhoea	7 (28)	8 (32)	7 (28)	22 (88)
Rash	11 (44)	3 (12)	0 (0)	14 (56)
Asthenia	5 (20)	6 (24)	2 (8)	13 (52)
Decreased appetite	6 (24)	5 (20)	1 (4)	12 (48)
Nausea	6 (24)	5 (20)	1 (4)	12 (48)
Rhinitis	6 (24)	0 (0)	0 (0)	6 (24)
Dry skin	5 (20)	0 (0)	0 (0)	5 (20)
Hypomagnesaemia	5 (20)	0 (0)	0 (0)	5 (20)
Mucosal inflammation	3 (12)	2 (8)	0 (0)	5 (20)
Muscle spasms	5 (20)	0 (0)	0 (0)	5 (20)
Skin fissures	5 (20)	0 (0)	0 (0)	5 (20)
Vomiting	2 (8)	2 (8)	1 (4)	5 (20)
Weight decreased	4 (16)	1 (4)	0 (0)	5 (20)
Dysphonia	4 (16)	0 (0)	0 (0)	4 (16)
Hypokalaemia	2 (8)	0 (0)	2 (8)	4 (16)
Rhinorrhoea	4 (16)	0 (0)	0 (0)	4 (16)
Dry mouth	3 (12)	0 (0)	0 (0)	3 (12)
Epistaxis	3 (12)	0 (0)	0 (0)	3 (12)
Hepatocellular injury	1 (4)	0 (0)	2 (8)	3 (12)

Abbreviations: AE=adverse event; NCI-CTCAE=National Cancer Institute Common Terminology Criteria for Adverse Events.

[a]No Grade 4 or 5 treatment-related AEs were observed.
