# Supplementary material for: Phase I study of afatinib combined with nintedanib in patients with advanced solid tumours
Source: Br J Cancer. 2015 Oct 29;113(10):1413–20. doi: 10.1038/bjc.2015.374 (PMC4815889; doi:10.1038/bjc.2015.374)
Supplement: Supplementary Table 2 [file bjc2015374x3.pdf]

Supplementary Table 2. Exploratory CTC analyses in patients with epithelial tumours in the dose-escalation phase, according to tumour response[a]

	Day 0	Day 15	Day 30	Day 60
**All patients (*n* = 40)[b]**				
CTC samples, *n*	39	37	34	21
Patients with <5 CTC, *n* (%)	29 (74)	31 (84)	30 (88)	16 (76)
Patients with ≥5 CTC, *n* (%)	10 (26)	6 (16)	4 (12)	5 (24)
**Patients with PR or SD (*n* = 26)**				
CTC samples, n	25	25	25	21
Patients with <5 CTC, *n* (%)	19 (76)	20 (80)	22 (88)	16 (76)
Patients with ≥5 CTC, *n* (%)	6 (24)	5 (20)	3 (12)	5 (24)
**Patients with PD (*n* = 9)**				
CTC samples, *n*	9	9	8	0
Patients with <5 CTC, *n* (%)	6 (67)	8 (89)	7 (87)	-
Patients with ≥5 CTC, *n* (%)	3 (33)	1 (11)	1 (13)	-

Abbreviations: CTC=circulating tumour cells; PD=progressive disease; PR=partial response; SD=stable disease.

[a]Results expressed as number of CTC per 7.5 mL of blood. Favourable: <5 CTC; unfavourable: ≥5 CTC.

[b]Five patients with tumours of non-epithelial origin (one patient with Ewing sarcoma and four patients with melanoma) were not included in the analysis.
